# Semen Analysis in “Urology-Naïve” Patients: A Chance of Uroandrological Screening in Young Males

**DOI:** 10.3390/jcm12113803

**Published:** 2023-06-01

**Authors:** Stefano Puliatti, Stefano Toso, Marco Ticonosco, Salvatore Rabito, Maria Chiara Sighinolfi, Riccardo Ferrari, Vincenzo Rochira, Daniele Santi, Tommaso Trenti, Michele Navarra, Stefania Ferretti, Luigi Montano, Salvatore Micali

**Affiliations:** 1 Department of Urology, University of Modena e Reggio Emilia, 41125 Modena, Italy; stefano.puliatti@unimore.it (S.P.);; 2 Unit of Endocrinology, Department of Medical Specialties, Azienda Ospedaliero-Universitaria of Modena, 41121 Modena, Italy; 3 Toxicology and Advanced Diagnostics, Ospedale S. Agostino-Estense, 41126 Modena, Italy; 4 Department of Chemical, Biological, Pharmaceutical and Environmental Sciences, University of Messina, 98166 Messina, Italy; 5 Andrology Unit and Service of Lifestyle Medicine in Uroandrology, Local Health Authority (ASL) Salerno, Coordination Unit of the Network for Environmental and Reproductive Health (EcoFoodFertility Project), Oliveto Citra Hospital, 84124 Salerno, Italy; 6 PhD Program in Evolutionary Biology and Ecology, University of Rome “Tor Vergata”, 00133 Rome, Italy

**Keywords:** semen analysis, screening, young male patients, infertility, pollution, EcoFoodFertility project

## Abstract

(1) Background: While females start their gynecological examinations during puberty, only few men decide to be visited by urologists in their youth. Given the participation in the EcoFoodFertility research project, our department had the opportunity to screen young males that were supposedly healthy. (2) Results: from January 2019 to July 2020, we evaluated 157 patients with sperm, blood analysis, and uroandrological examinations. The inclusion criteria were age 18–40 and absence of previous urological disease (urology-naïve). The primary endpoint of the study was to record uroandrological diseases that are occasionally discovered during examination in asymptomatic young men. The average age was 26.9 years (range 18–40); average testicular volume was 15.7 mL (range 12–22 mL); and 45.2% reported abnormal semen analysis: 62 cases of teratozoospermia, 27 asthenozoospermia, 18 oligozoospermia, and 2 azoospermia were discovered respectively; 4/157 patients were diagnosed with hypogonadism; 2 cases with suspicious testicular mass resulted in testicular cancer; and 31 suspected varicoceles and 8 patients with mild sexual dysfunctions were managed. (3) Conclusions: an uroandrological evaluation of young asymptomatic males allowed for the prompt diagnosis of different urological conditions, including cancerous ones, in our series. Despite being debatable, combining urological counselling with physical examination, semen analysis, and a laboratory profile could be useful and cost-effective in order to ameliorate male health.

## 1. Introduction

Currently, the first examination of urological screening is recommended around the age of 50 [1]; in clinical practice, even though it is common for the female population to undergo a gynecological examination during their pubertal phase, it is not common for young male patients to be visited by a urologist even if there are many typical urological diseases that arise around that age, such as varicoceles, sexual-related infection, infertility, and testicular cancer [2,3]. Moreover, the progressive increase of male infertility in the last decades [4,5] and the fall of semen quality not only across western countries [6,7] indicates how important it is to carry out andrological prevention at a young age by performing not only clinical evaluation but also semen analysis.

Relevance and clinical attention have been given to the evaluation of general health in relation to the male reproductive system: unfertile patients are more likely to be unhealthy regarding their general status compared to a fertile one, given that poor semen parameters and a diagnosis of male infertility are associated with an increased risk of hypogonadism, cardiometabolic disease, diabetes, blood hypertension, cancer, reduced life expectancy, and even mortality [8,9,10].

Furthermore, it is important to protect gametes in preconception and particularly at a young age to reduce the risk of epigenetically transmitted diseases to future generations [11].

Our center is taking part in a multicenter study based in Salerno, termed the EcoFoodFertility project (approved by the Ethical Committee of the Local Health Authority Campania Sud-Salerno, Committee code n. 43 of 30 June 2015, https://www.ecofoodfertility.it/, accessed on 12 July 2022), whose aim is to evaluate the role of human semen as an early biomarker of pollution in healthy men living in various areas with different environmental impacts. So far, the preliminary results support the belief that human semen is a sentinel biomarker of environmental exposure [12,13,14,15,16,17]. More surprisingly, semen quality and sperm count show differences across various areas of the same country or region, thereby supporting the idea that environmental factors are primarily responsible for these results [13,14,17]. On the other hand, several studies report that in heavily polluted areas, there is an increase in infertility, urogenital malformations, and chronic diseases (cancer, diabetes, etc.) [17]. On the basis of these considerations, and in particular following a recent Italian study, which found that areas with a high environmental impact had reduced semen quality in healthy young people [17], considering the Modena area, according to the Regional Environmental Agency of Emilia Romagna Region, with significant rates of air pollution, the study on these young boys appears to be an important measure of andrological prevention and general health considering the quality of the human semen as an important indicator of both environmental and general health [13,14,15,16].

First-level exams that were required for this study were semen analysis, a simple, easy, and cheap exam, blood analysis, and uroandrological examinations in urological naïve and healthy patients, who had never undergone a urological examination prior to this study.

## 2. Materials and Methods

We examined 157 patients from January 2019 and July 2020, with sperm analysis, blood analysis, and uroandrological examination. All methods were carried out in accordance with the Code of Ethics of the World Medical Association (Declaration of Helsinki) guidelines and regulations [18]. The study has been approved by the local ethic committee (Area Vasta Emilia Nord, prot. n. 0014975/18). All included patients signed their informed consent to participate in the study.

Parameters to participate in the study were being between 18 to 40 years of age, having no previous urological disease, and being a non-smoker. All patients lived in our area (Modena and the surrounding province). A sexual abstinence of 3 to 5 days was necessary before collecting the semen.

Urologists collected the age, height, body mass index (BMI), waist, and hip circumference of all the included patients during physical examination.

Semen analyzes were performed according to the World Health Organization (WHO) 2010 guidelines [19]. All samples were analyzed with the optical microscope LEICA DM2000LED by an expert biologist.

Blood examinations were conducted to collect TSH, FSH, LH testosterone, estradiol, hemoglobin, white blood cells, creatinine, ALT, AST, G-GT, total cholesterol, and triglycerides (as shown in Table 1 and Table 2).

All patients underwent an andrological physical examination, evaluating any abnormalities present in the external genitalia and reporting the testicular volume.

Accurate uroandrological clinical histories were collected for every patient, and each of them answered two questionnaires about their physical activity, and diet habits related to their recent past; the questionnaires, supplied by EcoFoodFertility project, were all validated prior to being handed out to the patients.

All methods were carried out in accordance with the relevant guidelines and regulations.

## 3. Results

Anthropometric measurements of the included patients in this study revealed an average age of 26.9 years (range 18–40 years), and an average testicular volume of 15.7 mL (range 12–22 mL), among other characteristics, as shown in Table 3.

A total of 157 semen analyzes (one conducted per patient) were performed in this study. Two patients resulted in azoospermia; 18 in oligozoospermia (11.5%); 27 in asthenozoospermia (17%); and 62 in teratozoospermia (39.5%), respectively.

Moreover, 45.2% of the patients resulted with at least one altered seminal parameter (Table 4).

Blood analysis was also performed for all 157 patients in this study: four patients with suspected hypogonadism, classified as having a serum testosterone level below 3 ng/mL, were identified as a result.

All subjects then underwent their physical examination, and 31 suspected varicoceles were found during these physical examinations.

Two patients with a suspected testis nodularity were sent for further assessments, which have since revealed a testicular cancer prognosis. No patients reported being involved in any pregnancy until the study, and eight patients reported to have experienced sexual dysfunctions that were deemed to be anxiety-related.

Importantly, no statistically significant results were found in analyzing the correlation between the lifestyle and diet habits questionnaires.

## 4. Discussion

Most young patients are either unaware that there is a definite medical field for treating uroandrological diseases, or that they simply feel uncomfortable talking about andrological issues.

Moreover, most of the young patients do not have a specific adult figure to whom they can rely on about sexual education and sexual health.

Alteration of fertility or infertility is an important, but underrated health problem among the young male population, although it is well known nowadays that the existence of reduced fertility can present even at a young age. [20]. In some cases, it has been attributed to urological problems, while in other cases it can arise either due to endocrinological issues, or for reasons still unexplained [21].

A recent systematic review [22] analyzed the lack of awareness of young patients regarding uroandrological diseases, assessing the importance of clinicians in instructing young men to familiarize themselves with urological issues (i.e., testicular self-examination).

Following our participation in the multicentric study “EcoFoodFertility” and the consequent sampling of seminal fluid and blood tests from each patient attending the study, we carefully analyzed the results. What we found was particularly interesting, especially related to the fact that all patients were “urology naive”, volunteers, and asymptomatic.

Concerning fertility, only 54.8% of all patients presented semen analysis with all normal parameters; the remaining population turned out having alterations in motility, morphology, or concentration of spermatozoa according to the WHO parameters (WHO 5th Edition). Furthermore, normozoospermia does not necessarily indicate fertility. Reference values defined by WHO have changed over the decades, lowering their parameters through the years [23].

An interesting meta-analysis, which collected data from 1981 until 2013, revealed a progressive reduction of male fertility through the years [20]. The authors collected data from studies that referred to male infertility related to any kind of pathological disorder.

The main difference in our study was regarding the selection of patients, following the indication of the pilot project, with each one of our patients being “uroandrological naive” (defined as not previously aware of andrological problems).

This selection helped us to identify patients with a prominent alteration in semen analysis, and to give them a medical treatment and further tests to better frame their problem, with cryopreservation even being considered.

However, it is still not clear how to manage asymptomatic patients who present with a mild alteration of the spermiogram, and who are not actively trying to have children [24].

Testicular dysgenesis syndrome can be an underlying factor for this group of patients with mild alterations, as exposure to environmental contaminants, birth impairments, and fetal alterations seem to have a role, but the main reasons still remain unclear [25]. Further studies should be headed in this direction.

No correlations between the daily habits (such as assumptions of drugs or lifestyle) or physical characteristics and spermiogram results were found in the population; in the literature, similar results have been reported [26,27].

Patients diagnosed with a suspected left varicocele at the physical examination received the indication of a testicular ECD-US to eventually confirm their diagnosis and begin their surgical treatment.

As suggested by the EAU guidelines, surgical treatment is essential in varicoceles presenting with alterations on semen analysis [28,29].

To prevent the overtreatment of varicoceles, the availability of semen analysis and physical examination (all patients who underwent surgery also showed a reduction of testis volume) allows urologists to promptly direct patients to the correct diagnosis and therapeutic choice.

Aside from the fertility patterns observed, the remarkable (and casual) detection of two testicular cancers were identified from this study; these patients already knew about their nodularity but underestimated its importance.

This result may be surprising, but not unexpected, especially considering testicular cancers are a type of tumor that are known to affect young men [30]; moreover, male infertility was associated with a subsequent elevated risk of testicular cancer [31].

Both of these patients underwent orchifunicolectomy; a classic seminoma and a mixed non-seminoma tumor were confirmed at the definite histology. They are now undergoing oncological follow-up.

Four patients were found with possible hypogonadism following hormonal analysis, and they were sent for further endocrinological investigation.

Erectile dysfunction (ED) in the young population is recently gaining attention; it is reported in up to 25% of patients from 18 to 40 years who have suffered at least one episode of ED in their lifetime [32]; an imperative is to define the eventual organic or psychological cause [33].

Moreover, the widespread use of pornography could have a role [34] in young patients suffering ED, especially in a psychological-related manner.

The role of andrological visits in psychogenic ED has been described in the literature [35].

Among our patients, eight males had the opportunity to discuss their sexual issues (mild erectile dysfunction related to anxiety pattern) for the first time with a qualified figure, finally setting aside their embarrassment. In this case, no pharmacological treatment was necessary, only behavioral therapy or a modification of their lifestyle were deemed sufficient, based on their precise medical history. Having the chance to talk to a specific professional figure about their “taboo” questions helped with their therapy and allowed the resolution of the sexual matter.

The limitations in our study were associated with the risk of overdiagnosis in impaired fertility: at the moment of the analysis, a few patients have since wanted to have children; however, for a patient, this could lead to them being in an anxiety-induced state even when they are not actively trying to have babies or do not have the diagnosis of couple infertility.

Our study is still ongoing and increasing the numerosity could further confirm our hypothesis of a general urological screening in young male patients.

In our opinion, the cost of a semen analysis (aside from blood and hormonal analysis, which could be performed as an II-level evaluation), together with an uroandrological examination could be sustainable for the sanitary system, and even more if it could lead to a general adjustment of uroandrological health in patients who otherwise would not be aware about their condition and the pathologies they suffer from.

## 5. Conclusions

In our experience, for young patients, a urological examination accompanied with semen analysis, could be useful to safeguard general urological health and may allow young men to experience the existence of a reference figure for the approach to their fertile sexual life. The use of the spermiogram permits fertility evaluation, and could confirm and better frame any associated co-pathologies, thanks to its low cost and simplicity of execution. Further studies focusing on large-scale feasibility and on the eventual assessment of the role of testicular dysgenesis syndrome are needed.

## Figures and Tables

**Table 1:** Hormone analyzes comprising all the patients enrolled.

	Number	Minimum	Maximum	Mean	Standard Deviation
TSH (microIU/mL)	157	0.05	70.0	2.2	5.4
FSH (IU/L)	157	1.0	60.7	3.9	4.9
LH (IU/L)	157	1.0	13.3	3.6	1.6
Testosterone (ng/mL)	157	2.2	12.0	5.4	1.7
Estradiol (pg/mL)	157	5.6	60.0	21.4	7.5

**Table 2:** Blood routine analyzes of all included patients.

	Number	Minimum	Maximum	Mean	Standard Deviation
Hemoglobin	157	5.2	18.2	14.9	1.2
White blood cells	157	3.0	13.5	5.9	1.7
Creatinine	157	0.7	127.0	3.0	15.5
ALT	157	9	227	27.0	25.3
AST	157	12	469	32.7	51.1
G-GT	157	8	60	20.8	8.7
Total cholesterol	157	79	259	176.1	33.7
Tryglicerides	157	19	612	90.3	66.4

**Table 3:** Anthropometric characteristics of all enrolled patients.

	Number	Minimum	Maximum	Mean	Standard Deviation
Age (years)	157	18.0	40.7	26.9	3.8
Height (cm)	157	167	196	179.5	63.7
Weight (kg)	157	55	110	76.6	9.3
BMI (kg/m^2^)	157	18.4	35.1	23.8	2.5
Waist circumference (cm)	157	65	106	83.4	10.3
Hip circumference (cm)	157	65	120	84.3	12.8
Testicular volume (mL)	157	12	22	15.7	2.1

**Table 4:** Semen analyzes comprising all included patients.

	Number	Minimum	Maximum	Mean	Standard Deviation
pH	157	3.0	9.0	8.1	0.5
Volume	157	0.5	10.0	3.4	1.8
Sperm concentration	157	0	270.0	76.2	57.7
Total sperm number	157	0	770.00	229.7	179.8
Normal forms	157	0	20	4.3	2.8
Progressive motility	157	0	94	51.6	20.4
Total motility	157	0	97	58.5	19.7

## Data Availability

The data that support the findings of this study are available on request from the corresponding author [L.M.].
